# Anisotropic Thermal Expansion of Transparent Cellulose Nanopapers

**DOI:** 10.3389/fchem.2020.00068

**Published:** 2020-02-07

**Authors:** Takayuki Hirano, Kana Mitsuzawa, Shun Ishioka, Kazuho Daicho, Hiroto Soeta, Mengchen Zhao, Masaaki Takeda, Yoshihiro Takai, Shuji Fujisawa, Tsuguyuki Saito

**Affiliations:** ^1^Material Characterization Laboratories, Toray Research Center, Otsu, Japan; ^2^Department of Biomaterial Sciences, Graduate School of Agricultural and Life Sciences, The University of Tokyo, Tokyo, Japan

**Keywords:** cellulose nanofiber, thermal expansion, anisotropy, film, nanopaper

## Abstract

We report the anisotropic thermal expansion of a transparent nanopaper structure comprising cellulose nanofibers (CNFs). The coefficient of thermal expansion (CTE) of the nanopaper in the out-of-plane direction was 44.6 ppm/°C in the temperature range of 25–100°C, which is approximately five times larger than its CTE in the in-plane direction in the same temperature range (8.3 ppm/°C). Such a strong anisotropy in thermal expansion is mainly attributable to the anisotropic CTE values of single CNFs in the fiber axis and cross-sectional directions. We observed anisotropic thermal expansion even in a bioplastic composite containing only 2.5% w/w CNFs.

## Introduction

Cellulose nanofibers (CNFs) are high-performance biobased materials with high mechanical strength and low thermal expansion. They are produced as water dispersions and can be formed into films. The production of CNF dispersions and films is well-established, and their uses are now being explored. CNF films are often called “nanocellulose paper” or simply “nanopaper” (Henriksson et al., 2008; Zhao et al., 2018), because they are made from nanometer-wide fibers and form paper-like network structure at the nanoscale. Some transparent grades of the nanopapers are even called “transparent paper” (Nogi et al., 2009). The nanopaper structure can exploit the potential of CNFs, and combine mechanically and thermally superior properties.

A promising application of nanopapers is their use as flexible substrates in electronic devices (Nogi et al., 2009). In recent years, electronic devices have become smaller and thinner, and are often required to fit into very limited spaces. In such cases, it is important to reduce any thermal stress on the substrates. Therefore, substrates need high dimensional stability and favorable radiation performance. Single CNFs are thermally anisotropic. The thermal conductivity of nanopapers is significantly higher in the in-plane direction than in the out-of-plane direction (Diaz et al., 2014; Uetani et al., 2015). Such anisotropic conductivity could enable the design of new heat management for electronic devices (Uetani et al., 2017). The nanopapers also have a low coefficient of thermal expansion (CTE) in the in-plane direction (Nogi et al., 2009; Diaz et al., 2013), which makes them particularly useful as substrates. However, to the best of our knowledge the CTE of the nanopapers in the out-of-plane direction has not yet been reported.

Herein, we demonstrate the anisotropy in thermal expansion of transparent nanopapers. The nanopapers were prepared from a 2,2,6,6-tetramethylpiperidin-1-oxyl (TEMPO)-oxidized CNF/water dispersion (Zhao et al., 2018). The in-plane and out-of-plane CTE values of the nanopapers were determined by thermomechanical analysis (TMA) and laser interferometry, respectively. For reference, two other cellulosic films were assessed in the same manner: one was a bioplastic film of cellulose acetate (CA), and the other was a composite film with a CA matrix containing 2.5% w/w TEMPO-oxidized CNFs.

## Materials and Methods

### Materials

A 0.4% w/w TEMPO-oxidized CNF/water dispersion was prepared according to the method described in a previous report (Zhao et al., 2018). The carboxylate content, weighted-average length and width of the CNF sample were ~1.2 mmol/g, 1 μm, and 3 nm, respectively. The CNF dispersion (80 mL) was poured into a 90-mm-diameter polystyrene petri dish and dried at rest in an oven at 40°C for 1 week. The resulting nanopaper was peeled from the petri dish and then conditioned at 23°C and 50% relative humidity for more than 2 days. The CA (LT-35) was supplied by Daicel Corp., Tokyo, Japan. The degrees of polymerization and substitution of the CA are 270 and 2.87, respectively, according to the data catalog. A 2% w/w CA solution was prepared using *N, N*-dimethylacetamide (DMAc) as the solvent. The CA solution (25 mL) was poured into a 90-mm-diameter glass petri dish and dried at rest *in vacuo* at 40°C for 1 day and subsequently at 70°C for 1 week. The CA/CNF composite was prepared from a mixture of the CA solution (25 mL) and a 0.1% w/w TEMPO-oxidized CNF/DMAc dispersion (12 mL), according to the method described in a previous report (Soeta et al., 2017). The mixture (12 mL) was dried under the same conditions as for the CA film. The dried films of the CA and CA/CNF composite were peeled from the petri dishes and conditioned as well as the nanopaper.

### TMA

The TMA was performed using a Shimadzu TMA-50 system in a nitrogen atmosphere within a temperature range of ~25–120°C at a heating rate of 2°C/min. Before analysis, the film specimens (plane size 12 mm × 10 mm) were dried at 130°C in the device. The specimens were positioned vertically, and the dimensional changes in the in-plane direction were measured under a slight compression load (5 mN) on the cross-sections of the specimens.

### Laser Interferometry

The laser interferometry was performed using an Advance Riko LIX-2 system in a helium atmosphere at ~90 kPa under the same thermal conditions used for the TMA. The specimens (plane size 7 mm × 7 mm) were positioned horizontally, and their dimensional changes in the out-of-plane direction were measured under a slight compression load (167 mN; ~6 kPa) on the surfaces of the specimens.

### Scanning Electron Microscopy (SEM)

A rectangular specimen 3 mm × 30 mm in size was cut out from a nanopaper. The specimen was fractured by uniaxial tensile load using a Shimadzu EZ-SX at 23°C and 50% relative humidity. The gauge length and head speed were set to 10 mm and 1 mm/min, respectively. The fractured surface was treated using a Meiwafosis Neoc osmium coater at 6 mA for 2.5 s. The osmium-treated surface was observed by SEM using a Hitachi S-4800 at 1.5 kV.

### Raman Spectroscopy

Raman spectroscopy was carried out using a customized Photon Design near-infrared Raman spectrometer equipped with a YAG laser (wavelength 1,064 nm) and a Nippon Roper InGaAs detector. The intensities of the Raman band at 1,100 cm^−1^ (glycoside bond) were recorded as a function of the rotational angle of the polarization, as reported previously (Wanasekara et al., 2016).

## Results and Discussion

The nanopapers were prepared through evaporative condensation of a TEMPO-oxidized CNF/water dispersion. The TEMPO-oxidized CNFs are spontaneously arranged into a polydomain nematic order on the condensation process, such that a plywood-like nanostructure of the densified nematic-CNF arrangement is finally formed in the dried nanopapers. [See a reference by Zhao et al. (2018) for details of the nematic ordering.] The nanopapers were ~30-μm thick, and were optically transparent. The reference films of the CA and CA/CNF composite were prepared through solvent casting *in vacuo*, according to the method described in a previous report. In the composite, TEMPO-oxidized CNFs are homogeneously distributed at the ratio of 2.5% w/w and form a percolating network in the CA matrix. [See a reference by (Soeta et al., 2018) for details of the composite structure]. Both the CA-based films were ~80-μm thick, and were optically transparent as well as the nanopapers (Soeta et al., 2017).

**Figure 1:**
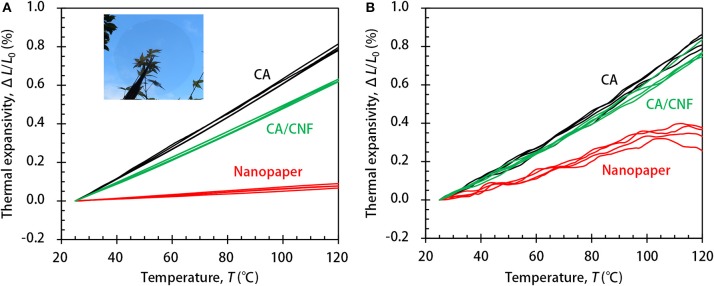
The thermal expansivity of the nanopaper and CA-based films in the **(A)** in-plane and **(B)** out-of-plane directions. The inset in **(A)** illustrates the optical transparency of the nanopaper.

Figure 1 shows the thermal expansivity Δ*L*/*L*_0_ of the nanopaper and CA-based films in the in-plane and out-of-plane directions, where *L*_0_ is the initial length or thickness of the specimen at ~25°C, and Δ*L* is the change in the corresponding dimension of the specimen following thermal expansion. The four measurements for each type are displayed in the figure and show the high reproducibility. The temperature dependency of the thermal expansivity was approximately linear in the temperature range Δ*T* of 25–100°C for all three types of specimen in both the in-plane and out-of-plane directions. At the temperature of ~100°C, only the expansivity of the nanopaper in the out-of-plane direction somehow showed an infection point, and became nearly flat in the higher Δ*T* of 100–120°C. This is perhaps related to the specific thermal expansion of wood cellulose crystallites; Hori and Wada (2005) observed that the hydrogen bonding sheet significantly shrank along the *b*-axis of the unit cell (−30 ppm/°C) although the sheet stacking distance of the *a*-axis expanded (136 ppm/°C). This interpretation should be based on the orientation of crystallites to the out-of-plane direction, which is described in the latter part of this paper. Note that not only CNFs but also the CA have no glass transition and melting points in the whole Δ*T* of 25–120°C (Soeta et al., 2018)

The CTE values were calculated from the linear regions in the Δ*T* of 25–100°C using the following equation:

$$\begin{array}{l} \bar{a}=\frac{1}{L_{0}}\cdot \frac{\Delta L}{\Delta T} \end{array}$$

The values are shown in Table 1 as the mean and standard deviation of the four measurements in Figure 1. The CTE of the nanopaper in the in-plane direction was as low as 8.3 ppm/°C, which is comparable to that of a glass, as previously reported (Nogi et al., 2009). In contrast, the CTE in the out-of-plane direction was significantly higher (44.6 ppm/°C). A CTE of 44.6 ppm/°C is still as low as that of a polymeric solid, but some grades of plastics have similar or even lower CTE values. The reference CA film was thermally isotropic as predicted, and its CTE values in the in-plane and out-of-plane directions were both ~80 ppm/°C. Whereas, the out-of-plane CTE of the CA/CNF composite was also ~80 ppm/°C, it is significant that its in-plane CTE value was lower (65.1 ppm/°C).

**Table 1:** The in-plane and out-of-plane CTE values of the nanopaper and CA-based films in the temperature range of 25–100°C.

**Sample**	**CTE (ppm/**^****°****^**C)**	
	**In-plane**	**Out-of-plane**
Nanopaper	8.3 ± 1.2	44.6 ± 3.7
CA	82.5 ± 1.7	84.5 ± 2.2
CA/CNF	65.1 ± 1.0	78.0 ± 3.0

Note that the in-plane and out-of-plane CTE values of the specimens were determined by TMA and laser interferometry, respectively. The Δ*L* resolutions for TMA and laser interferometry are ~100 and 2 nm, respectively; in fact, the measurements were performed in the detectable ranges. For instance, the lower limit of the out-of-plane CTE value of the nanopaper is estimated to be ~0.9 ppm/°C using the *L*_0_ and Δ*T* values of 30 μm and 75°C (25–100°C), respectively, which is sufficiently lower than the experimental value of 44.6 ppm/°C.

**Figure 2:**
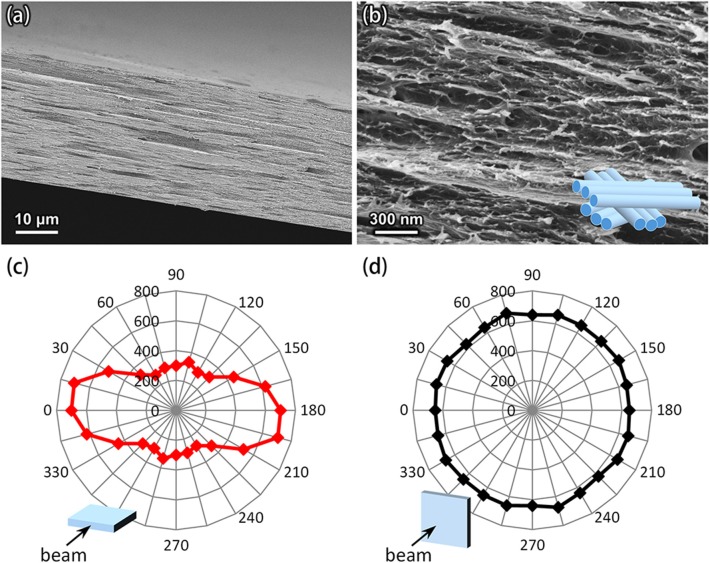
Structure of the nanopaper. **(a,b)** SEM images of a cross section of the nanopaper. The inset in the **(b)** shows a simplified model for the packing structure of CNFs. **(c,d)** Polar plots of the normalized intensities of the Raman band at 1,100 cm^−1^ (glycoside bond), recorded by applying the laser beam to **(c)** the cross-section and **(d)** the surface of the nanopaper.

It is interesting that the thermal expansion of transparent nanopapers was strongly anisotropic (~8:45). This phenomenon can be interpreted based on the thermal anisotropy of single CNFs (equal to the crystallites in structural meaning). The thermal expansion of individual CNFs in the fiber axis direction is strongly restrained by covalent bonds. The CTE of such fibers is estimated to be 6 ppm/°C in the case of wood cellulose (Hori and Wada, 2005). In contrast, the cross-sectional expansion of individual CNFs is governed by intermolecular interactions. The area CTE of the fiber cross-section is estimated to be 106 ppm/°C based on the data by Hori and Wada (2005). Thus, the mean linear CTE in the cross-sectional directions is calculated to be 53 ppm/°C, and is much larger than the axial CTE of 6 ppm/°C. The contribution of such thermal anisotropy of CNFs to the nanopaper expansion is realized by analysis of the nanopaper structure (see Figure 2).

Figure 2 shows how CNFs are packed in the nanopaper. The structure of nanopapers has multi-layered anisotropy (Figure 2a), and CNFs are packed approximately parallel to the in-plane direction of each layer (Figure 2b). This structure is supported by Raman spectroscopy for the fiber axis of CNFs (glycoside bond) in the nanopaper (Figure 2c). Also, such laid CNFs are isotopically stacked up to the out-of-plane direction (Figure 2d). XRD and FTIR analyses of the nanopaper are shown in Supplementary Figures S1, S2, respectively. [See also a reference by (Zhao et al., 2018) for nanopaper structure]. It is therefore reasonable to interpret the data in Table 1 as meaning that the CTE values of the nanopapers in the in-plane and out-of-plane directions mainly arise from those of individual CNFs in the fiber axis and cross-sectional directions, respectively. The anisotropy of the CA/CNF composite (~65:78) also suggests that the fiber axes of the CNFs in the CA matrix were on average perpendicular to the out-of-plane direction.

A similar anisotropy has been reported for the in-plane thermal expansion of uniaxially oriented cellulose nanocrystal (CNC) films (Diaz et al., 2013). The in-plane CTE values of the CNC films in the two directions parallel and perpendicular to the orientation axis are ~9 and 158 ppm/°C, respectively. The large in-plane CTE of 158 ppm/°C has been interpreted as the result including not only the cross-sectional expansion of crystallites but also the contribution of inter-crystallite (CNC–CNC) interfacial motion. However, the out-of-plane CTE of our nanopapers (44.6 ppm/°C) can only be explained by the cross-sectional expansion of crystallites (53 ppm/°C). This result indicates that the inter-CNF interactions are dominated by hydrogen bonding; as discussed above, the CTE of the hydrogen bonding sheet along the *b*-axis of the unit cell is nearly zero (Wada, 2002) or even negative (Hori and Wada, 2005), whereas the CTE of the hydrophobic sheet-stacking in the *a*-axis direction is as large as 43–136 ppm/°C. Note that XRD profiles of wood-derived CNC films and nanopapers show no orientation of the crystal planes to the film surface (Elazzouzi-Hafraoui et al., 2008; Daicho et al., 2018).

**Figure 3:**
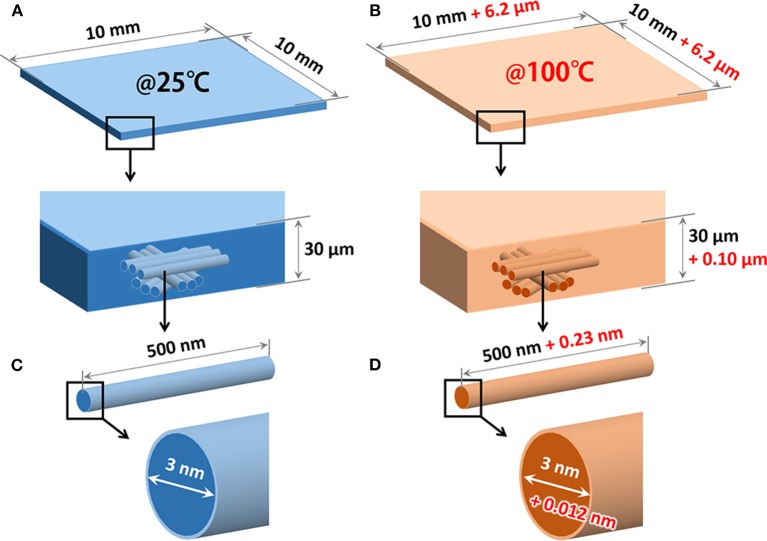
Association of the nanopaper expansion with thermal anisotropy of single CNFs. **(A,B)** Thermal expansion of a nanopaper specimen with dimensions of 10 mm × 10 mm × 30 μm in the temperature range of 25–100°C. The dimensional changes at 100°C (the values in red) are calculated using the CTE values in the in-plane (44.6 ppm/°C) and out-of-plane (8.3 ppm/°C) directions of the nanopaper. **(C,D)** Thermal expansion of a single CNF in the temperature range of 25–100°C. For simplification, the cross-sectional shape and length of the CNF are assumed to be a circle with a diameter 3 and 500 nm, respectively. The dimensions at 100°C (the values in red) are estimated using the reported CTE values in the fiber axis (6 ppm/°C) and cross-sectional (53 ppm/°C) directions of the single CNF.

Figure 3 summarizes our interpretation for the anisotropic nanopaper expansion. In the temperature range of 25–100°C, the out-of-plane CTE value (44.6 ppm/°C) was found to be larger than the in-plane value (8.3 ppm/°C); however, it should be noted that actual dimensional change in the out-of-plane direction for a thin nanopaper with a thickness of 30 μm is only 0.1 μm and is indeed much smaller than its in-plane change (6.2 μm on a side for a square 10 mm × 10 mm; Figures 3A,B). A similar remark holds for single CNFs (Figures 3C,D); the dimensional change in the cross-sectional direction for a single CNF with a diameter of 3 nm is estimated to be only 0.012 nm on average, which is much smaller than its length change (0.23 nm for a length 500 nm). The anisotropic CTE of nanopapers will be thus pronounced as actual dimensional changes for thick or laminated nanopapers. It is also worth mentioning that the dimensional change in the out-of-plane direction (~0.1 μm) is sufficiently larger than reported values of the root-mean surface roughness (~6–10 nm) for the nanopapers of TEMPO-oxidized CNFs prepared in the same manner as this work (Wu et al., 2014; Zhao et al., 2018).

## Conclusions

In the present study, we demonstrated the anisotropic thermal expansion of transparent nanopaper structures comprising CNFs. The out-of-plane CTE value of the nanopaper was approximately five times larger than the in-plane value (44.6 ppm/°C vs. 8.3 ppm/°C, respectively). Such a strong anisotropy in thermal expansion was mainly attributable to the anisotropic CTE values of single CNFs in the fiber axis and cross-sectional directions. It is interesting that a transparent polymeric solid exhibits such marked anisotropy in thermal expansion. Attention may be required when thick nanopapers are used in electronic devices; the nanopapers only have a favorably low CTE value in the in-plane direction. The bioplastic composite containing only 2.5% CNFs also exhibited anisotropy.

## Data Availability Statement

The datasets for this article are not publicly available because the raw data supporting the conclusions of this article will be made available by the authors, without undue reservation, to any qualified researcher. Requests to access the datasets should be directed to Takayuki Hirano, Takayuki_Hirano@trc.toray.co.jp, or Tsuguyuki Saito, asaitot@mail.ecc.u-tokyo.ac.jp.

## Author Contributions

TH, SF, and TS designed the experiments. SI and HS prepared the samples. TH and KM performed thermal analyses. KD performed XRD and FTIR analyses. MZ performed SEM observation. TH and TS mainly wrote the manuscript with the contributions of all the authors.

### Conflict of Interest

The authors declare that the research was conducted in the absence of any commercial or financial relationships that could be construed as a potential conflict of interest.
