# Supplementary material for: Anisotropic Thermal Expansion of Transparent Cellulose Nanopapers
Source: Front Chem. 2020 Feb 7;8:68. doi: 10.3389/fchem.2020.00068 (PMC7019374; doi:10.3389/fchem.2020.00068)
Supplement: Supplementary file 1 [file Data_Sheet_1.pdf]

Supplementary Material

Anisotropic Thermal Expansion of Transparent Cellulose Nanopapers

Takayuki Hirano*, Kana Mitsuzawa, Shun Ishioka, Kazuho Daicho, Hiroto Soeta, Mengchen Zhao, Masaaki Takeda, Yoshihiro Takai, Shuji Fujisawa, Tsuguyuki Saito*

*** Correspondence:**
Takayuki Hirano, Takayuki_Hirano@trc.toray.co.jp
Tsuguyuki Saito, asaitot@mail.ecc.u-tokyo.ac.jp

This file includes **Supplementary Figures S1 and S2**.

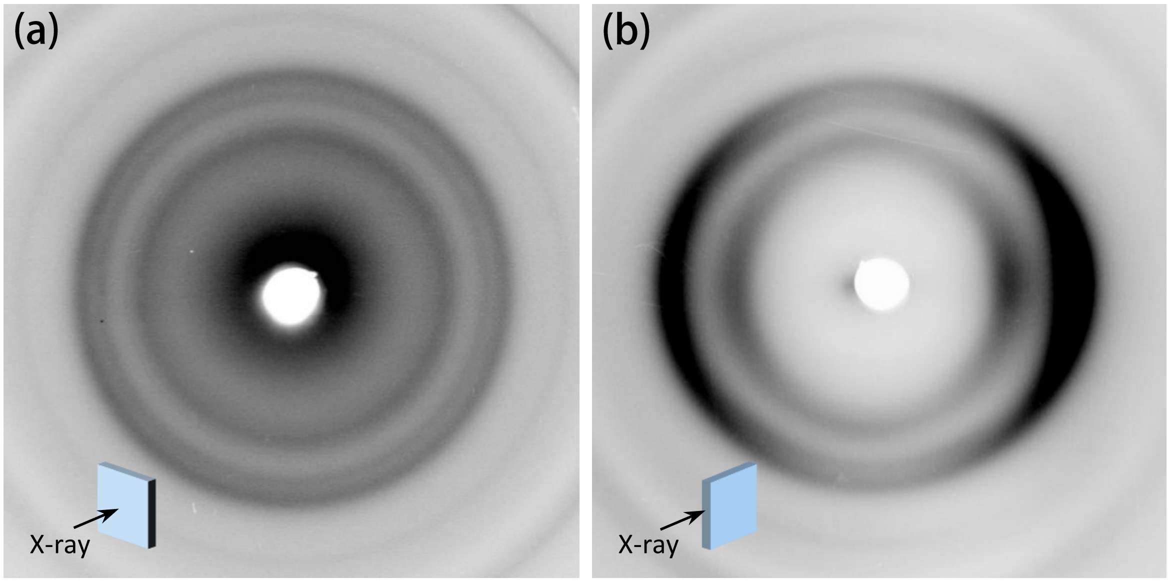

Supplementary Figure S1. XRD diagrams of the nanopaper for the incident beam (c) perpendicular or (d) parallel to the nanopaper surface.

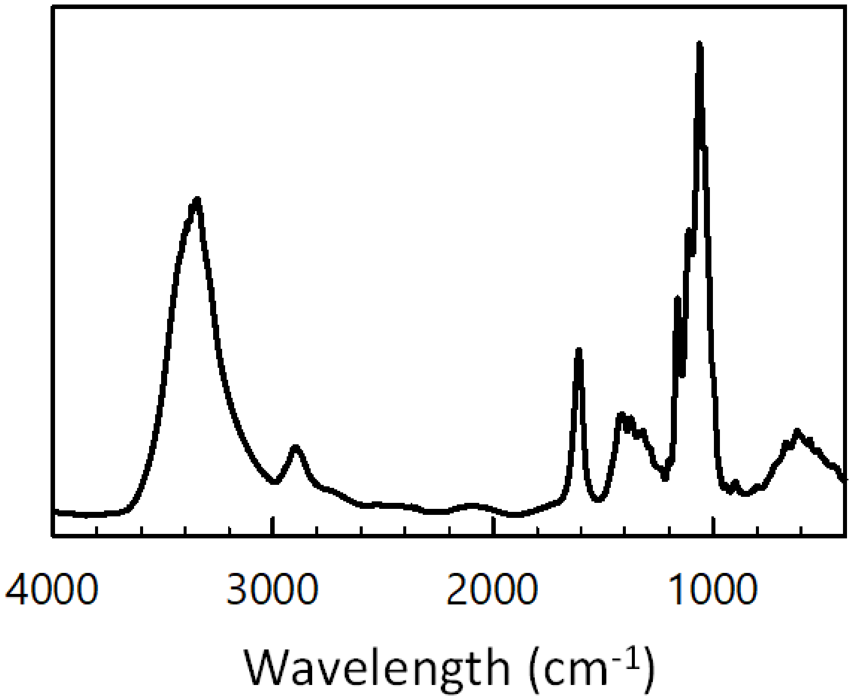

Supplementary Figure S2. FTIR spectrum of the nanopaper.
